# Three major effects of APOE^ε4^ on Aβ immunotherapy induced ARIA

**DOI:** 10.3389/fnagi.2024.1412006

**Published:** 2024-05-02

**Authors:** Kate E. Foley, Donna M. Wilcock

**Affiliations:** ^1^Stark Neurosciences Research Institute, Indiana University, Indianapolis, IN, United States; ^2^Department of Neurology, School of Medicine, Indiana University, Indianapolis, IN, United States

**Keywords:** ApoE4, ARIA, amyloid - beta, cerebral amyloid angiopathy (CAA), neuroinflammation, neurovascular unit (NVU)

## Abstract

The targeting of amyloid-beta (Aβ) plaques therapeutically as one of the primary causes of Alzheimer’s disease (AD) dementia has been an ongoing effort spanning decades. While some antibodies are extremely promising and have been moved out of clinical trials and into the clinic, most of these treatments show similar adverse effects in the form of cerebrovascular damage known as amyloid-related imaging abnormalities (ARIA). The two categories of ARIA are of major concern for patients, families, and prescribing physicians, with ARIA-E presenting as cerebral edema, and ARIA-H as cerebral hemorrhages (micro- and macro-). From preclinical and clinical trials, it has been observed that the greatest genetic risk factor for AD, APOE^ε4^, is also a major risk factor for anti-Aβ immunotherapy-induced ARIA. APOE^ε4^ carriers represent a large population of AD patients, and, therefore, limits the broad adoption of these therapies across the AD population. In this review we detail three hypothesized mechanisms by which APOE^ε4^ influences ARIA risk: (1) reduced cerebrovascular integrity, (2) increased neuroinflammation and immune dysregulation, and (3) elevated levels of CAA. The effects of APOE^ε4^ on ARIA risk is clear, however, the underlying mechanisms require more research.

## 1 Introduction

**Figure 1:**
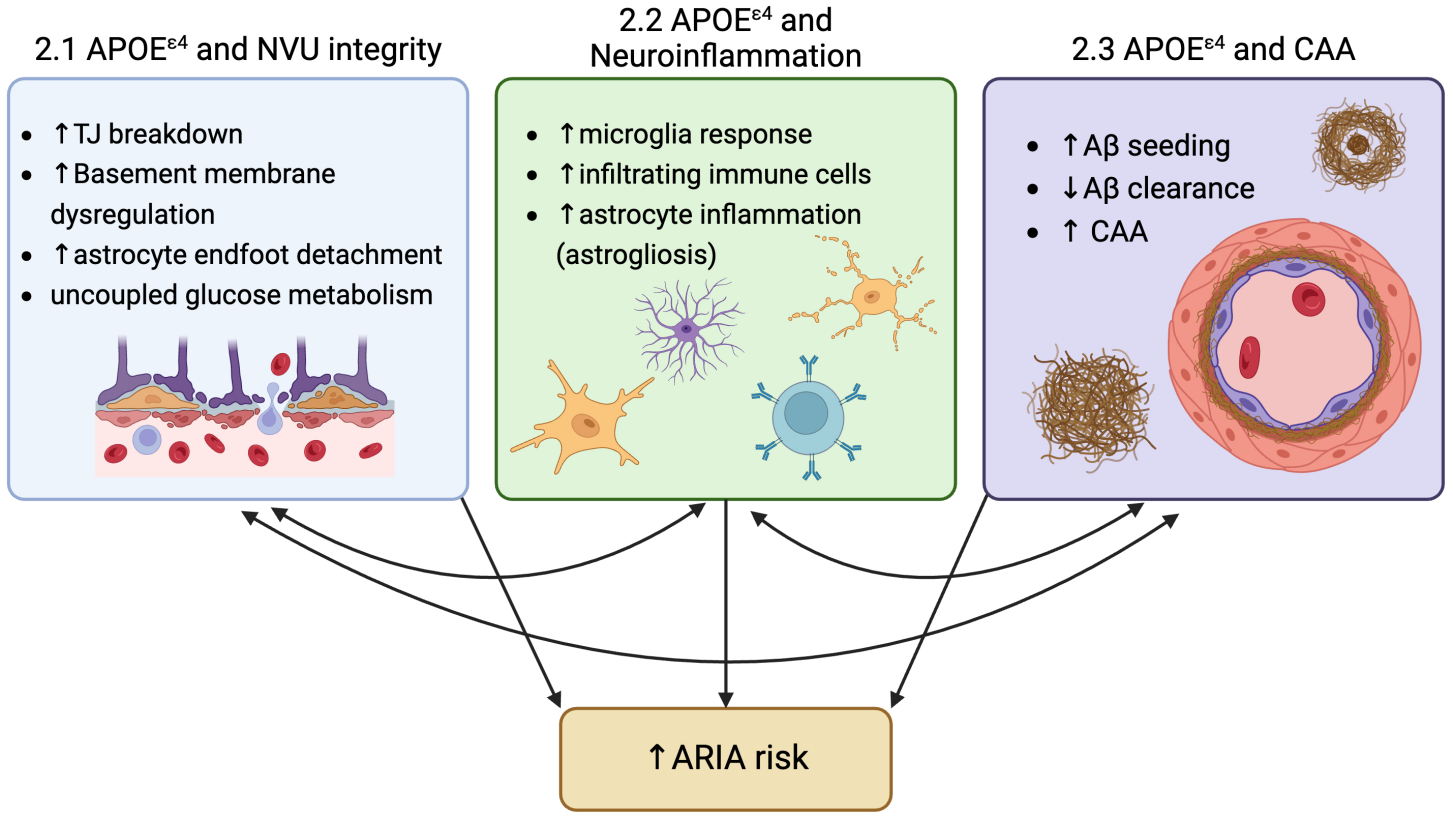
Three mechanisms by which ApoeE4 increase risk for ARIA: 1) Neurovascular unit (NVU) breakdown, 2) Increased neuroinflammation, and 3) increased amyloid-beta deposition and CAA..

For the first time since the description of Alzheimer’s disease (AD) in 1906, there is a class of drugs able to modify one of the two hallmark toxic protein aggregates in the brain that define AD. Decades of research into reducing amyloid-beta (Aβ) plaques produced disease-modifying therapies in the form of anti-Aβ antibody-based immunotherapy. As clinical trials on the safety, efficacy, and establishment of a ‘clinically meaningful’ reduction in cerebral Aβ have evolved, one common adverse side effect of anti-Aβ immunotherapies became a constant: Amyloid-Related Imaging Abnormalities, also known as ARIA. ARIA is detected by MRI and is classified in two forms, ARIA-E for edema, or ARIA-H for hemorrhage. This edema and hemorrhage (micro- and, on rare occasions, macro-) due to anti-Aβ antibody immunotherapy has been consistently observed across anti-Aβ antibody target residues and Aβ forms (Budd Haeberlein et al., 2022; Salloway et al., 2022; Van Dyck et al., 2022; Sims et al., 2023). While the collective hope for this class of drugs is palpable among researchers, clinicians, and patients, ARIA remains an ‘elephant in the room’ in the rollout of these drugs to AD patients. However, analyses of clinical trial results suggest two main contributors to ARIA are (1) increased anti-Aβ antibody dosage, and (2) carriage of the greatest genetic risk factor for AD, APOE^ε4^ (Figure 1).

This review focuses on the effects of APOE^ε4^ genotype on ARIA risk. APOE^ε4^ participants in clinical trials show much higher rates of ARIA incidence and increased severity of these events, with a homozygous APOE^ε4^ genotype at the greatest risk. In aducanumab clinical trials ENGAGE and EMERGE, ARIA-E was reported to be 29.5, 37.9 and 43.0% in APOE^ε4^ carriers at the low, medium and high doses, compared to none reported at the low dose, 17.8, and 20.3% at the medium and high dosages for APOE^ε4^ non-carriers (Salloway et al., 2022). For lecanemab, an FDA approved treatment, clinical trials revealed rates of 10.9% ARIA-E in APOE^ε4^ heterozygous carriers, and 32.6% in APOE^ε4^ homozygous carriers, compared to 5.4% in APOE^ε4^ non-carriers with lecanemab treatment (Van Dyck et al., 2022). Similarly, ARIA-H showed rates of 39.0% in APOE^ε4^ homozygotes and 14.0% in APOE^ε4^ heterozygotes compared to 11.9% in APOE^ε4^ noncarriers (Van Dyck et al., 2022). Donanemab clinical trials reported ARIA-E in 40.6% of the APOE^ε4^ homozygous participants, 22.8% in heterozygous APOE^ε4^ carriers, and 15.7% in APOE^ε4^ noncarriers (Sims et al., 2023). The stepwise incidence of ARIA due to APOE^ε4^ allele carriage has been demonstrated universally across trials of anti-Aβ antibody immunotherapy, however the mechanisms by which APOE^ε4^ causes this increased risk for ARIA is only starting to be unveiled.

APOE is a 34kD lipoprotein with two point mutations that designate the specific APOE allele at residues 112 (Cys, Arg) and 158 (Cys, Arg). These alleles predispose for AD in different manners, with APOE^ε3^ as the neutral benchmark for AD risk, APOE^ε2^ as reduced risk and APOE^ε4^ carriers exhibiting increased risk with up to a 3–4 fold increased risk of AD for one copy of APOE^ε4^, and 9–15 fold increased risk of AD with two copies of APOE^ε4^ (Yamazaki et al., 2019). It is known that the major function of APOE is cholesterol and lipid transport both in the periphery and the brain, however, there are many other mechanisms by which APOE predisposes the brain to AD pathology and as this review will highlight, ARIA.

APOE is primarily made by the liver for use in the periphery and by astrocytes in the brain, because APOE is not thought to cross the blood brain barrier, leaving two separate compartments of APOE in the body under unique regulation. During stressed conditions, many cell types are known to upregulate APOE production in the brain, and recently, multiple studies have identified mechanisms by which APOE provides a detrimental effect, with the reduction of microglial or neuronal APOE favoring a protective effect towards several dementia pathologies (Koutsodendris et al., 2023; Liu et al., 2023; Yin et al., 2023). In AD, those with the APOE^ε4^ allele showed lower levels of APOE abundance in the plasma (Gupta et al., 2011). Further, other studies have shown that rates of conversion from cognitively normal to either MCI or AD was significantly increased in each decade starting with age 60, with the largest hazard ratio of conversion due to APOE^ε4^ carriage being between ages 70–80 years old (Bonham et al., 2016). While there are many avenues being evaluated to elucidate the connection between the APOE^ε4^ allele and AD, we seek to highlight three mechanisms by which APOE^ε4^ increases risk for ARIA: (1) APOE^ε4^ and its effects on the neurovascular unit, (2) APOE^ε4^ and its effects on neuroinflammation, and (3) APOE^ε4^ and its effects on Aβ deposition.

## 2 Three mechanisms by which APOE^ε4^ increases risk for ARIA

### 2.1 APOE^ε4^ causes neurovascular unit (NVU) dysfunction

Under normal conditions, the neurovascular unit comprised of endothelial cells, pericytes, basement membrane, and astrocytes, maintains a delicate and tightly regulated signaling network for communication between neurons and the blood. The proper regulation of the blood brain barrier among these cells and matrices is critical to brain health. Edema, exchange of proteinaceous fluid from blood into the brain, or hemorrhage, leakage of whole blood into the brain, are both extremely toxic and can lead to wrought neurodegeneration. Evidence shows patients with previous microhemorrhages prior to anti-Aβ antibody treatment showed higher rates of ARIA (Withington and Turner, 2022). This correlation, combined with the data that APOE^ε4^ has been shown to elicit destructive effects on multiple components of the neurovascular unit, we propose is a major predisposition to ARIA when further challenged with an anti-Aβ antibody.

There is ample evidence that APOE^ε4^ influences the NVU integrity though multiple mechanisms. Studies in humans have shown APOE^ε4^-associated NVU dysfunction and worsened outcomes after major vascular events (Yip et al., 2005; Louko et al., 2006; Halliday et al., 2016; Montagne et al., 2020; Alruwais et al., 2022). Additionally, *in vitro* models show that APOE^ε4^ iPSC endothelial cells develop a leaky barrier through increased inflammatory cytokines, and overexpression of VWF, which is a prothrombotic inflammatory protein. Additionally, multiple reports show that APOE^ε4^ derived from astrocytes affects endothelial cell integrity though reduced tight junction coverage, directly impairing the barrier, and resulting in increased leakage through the endothelial cell monolayer (Nishitsuji et al., 2011; Jackson et al., 2022).

APOE^ε4^ also exhibits many effects to various APOE receptors and cascades, resulting in blood brain barrier breakdown. One APOE receptor, LRP1, is present on pericytes and has high binding of APOE^ε4^. LRP1 has been shown to signal through MMP9, a matrix metalloprotease that degrades a protective matrix around the neurovascular unit called the basement membrane as well as reduces endothelial tight junction proteins (Casey et al., 2015; Jackson et al., 2022). Impairment in pericyte migration is also a result of APOE^ε4^, with one finding showing that APOE^ε3^ can contribute to pericyte motility, however, APOE^ε4^ cannot (Casey et al., 2015). Pericyte produced APOE^ε4^ also reduces the ability for endothelial cells to form proper vessel shape, dysregulates basement membrane production signaling, and overall increases leakage (Yamazaki et al., 2020). Additionally, APOE^ε4^ is associated with reduced astrocytic end-foot connections to the cerebrovasculature, which reduces critical communication of neural needs and vessel integrity (Jackson et al., 2022). In addition to the extracellular effects of APOE^ε4^ on blood brain barrier integrity, evidence also suggests that the APOE^ε4^ genotype influences mitochondrial-metabolic health and glucose utilization, providing another avenue by which astrocytes, pericytes, and endothelial cells are functionally impaired (Farmer et al., 2021). New studies in mouse models suggest APOE^ε4^ driven neurovascular uncoupling, with APOE^ε3/ε4^ mice and APOE^ε4/ε4^ mice showing differential cerebral perfusion and glucose uptake (Onos et al., 2023). Further, boarder association macrophages (BAMs) have been shown to provide APOE^ε4^ to a detrimental effect to the neurovascular unit (Iadecola et al., 2023). When APOE is knocked out of BAM cells specifically, function of the neurovascular unit is rescued, providing another cell type and mechanism by which APOE regulates NVU integrity.

One of these dysfunctions alone could predispose the critical blood brain barrier to leakage of proteinaceous fluids or whole blood. If there is existing blood brain barrier compromise at the neurovascular unit, it may remain undetected by MRI if the effects are microscopic, however macro effects may show up as baseline edema, hemorrhages, or other stroke symptoms. Layering on an anti-Aβ antibody immunotherapy has been thought to exacerbate these existing effects, as well as produce new blood brain barrier breakdown events. Baseline MRIs as well as cardiovascular risk factor assessments, are two efforts that have been made in clinical trials, and now in clinical practice, to identify participants that may have existing NVU dysfunction leaving them at an increased risk for ARIA. However, while these efforts are applied across genotypes, it is clear from decades of research that APOE^ε4^ carriers have an existing heightened risk for ARIA due to the multiple mechanisms by which APOE^ε4^ disrupts the NVU integrity.

Taken together, there are many opportunities for APOE^ε4^ mediated neurovascular unit compromise, which over the course of a lifetime can accumulate. These deficits may result in large macro-events, presenting as strokes, or accrue over years with no clinically overt symptoms. Further, this predisposition for blood brain barrier dysfunction is one manner that leave an APOE^ε4^-carrying AD patients predisposed to ARIA when challenged with anti-Aβ antibody administration.

### 2.2 APOE^ε4^ and inflammation

In addition to the effects of APOE^ε4^ on multiple cells in the neurovascular unit, APOE^ε4^ also has been extensively shown to dysregulate the immune response (Vitek et al., 2009; Gale et al., 2014; de Leeuw et al., 2022; Mhatre-Winters et al., 2023). Microglia, the most abundant resident immune cell in the brain, can express APOE in response to stress, but can also respond to APOE signaling (de Leeuw et al., 2022). Human post-mortem studies have shown that those carrying APOE^ε4^ have different neuroinflammatory profiles through reactive microglia and proinflammatory cytokine expression (Egensperger et al., 1998; Overmyer et al., 1999; Friedberg et al., 2020). Some report elevated levels of reactive microglia in APOE^ε4^ carriers, while other studies show a blunted microglial response (Egensperger et al., 1998; Fitz et al., 2021; Iannucci et al., 2021; Kloske et al., 2021). However, the effects of microglial response having beneficial or detrimental effects on cognition are still controversial (Minett et al., 2016). Proinflammatory cytokines, TNFα, IL6, and IL1β are increased in primary mouse astrocytes with APOE^ε4^ expression when compared to APOE^ε3^ expression (Mhatre-Winters et al., 2023). It is suggested that this increased baseline level of TNFα in APOE^ε4^ carriers is a method of inflammatory predisposition, which in the short term may prove helpful to insults, however when chronically activated may cause constant neuroinflammation and immune exhaustion (Lanfranco et al., 2021). While microglia are the main immune cell, astrocytes can also upregulate inflammatory signals, with cell culture models showing increased inflammatory response in astrocytes in response to IL1b stimulation, which as previously mentioned is upregulated in APOE^ε4^ microglia (de Leeuw et al., 2022). APOE^ε4^ expression by astrocytes also increases astrocyte and microglia gliosis response in mouse models (Liu et al., 2017). APOE^ε4^ also networks with TREM2 on microglia, which triggers a neuroinflammatory response as well as phagocytosis of neurons (Atagi et al., 2015; Krasemann et al., 2017).

Neuroinflammation has long been associated across multiple attempts of Aβ clearance trials for AD modification. A clinical trial in the early 2000’s utilized active immunization of full length Aβ peptide, which was found to properly reduce Aβ plaques, however the trial was prematurely halted due to ~6% of participants acquiring meningoencephalitis. Studies on these patients revealed abnormal T-cell infiltration, microglial activation, and increased macrophage infiltration and engagement with some aspects resembling ARIA (Nicoll et al., 2003; Orgogozo et al., 2003; Pride et al., 2008). Similarly, bapineuzemab, the first passive administration of anti-Aβ antibody, showed increased vasogenic edema in phase II and phase III clinical trials leading to its termination, which was more prevalent in APOE^ε4^ carriers. The cause of the edema was unknown, however it became clear that APOE^ε4^ interacted with passive anti-Aβ antibody immunotherapies like aducanumab, lecanemab, and donanemab, when multiple clinical trials showed increased rates and severity of ARIA in APOE^ε4^ carriers. These two clinical trials together suggest a role of both APOE^ε4^ genotype and immune cell involvement in negative side effects of Aβ clearance. Interestingly, efforts to diminish microglial engagement with other immunotherapies like solanezumab (binds only soluble Aβ, thus not engaging central Fcγ receptors) and crenezumab (an antibody with an IgG4 backbone to minimize effector cell engagement due to low Fcγ receptor affinity) did not induce ARIA but also did not effectively lower brain amyloid burden. Thus, ARIA appears to involve effector cells, but efforts to mitigate this have also diminished efficacy, both with respect to clinical measures and amyloid burden. While ARIA incidence is not exclusive to APOE^ε4^ carriers and is also seen in APOE^ε3^ carriers, it is widely understood that the neuroinflammatory response may be playing a role in altering the rates of ARIA across APOE genotypes. Consistent findings of ARIA across antibodies that lower amyloid suggest there is a common mechanism by which these antibodies trigger more severe cerebrovascular dysfunction and increased neuroinflammation is a common affliction.

There have been multiple hypotheses that detail one major cause of ARIA stemming from neuroinflammation. One hypothesis presented at AAIC in 2023 suggests engagement of the classical complement cascade, which may further be influenced by the APOE^ε4^ allele (Rogers, 2023). Others have suggested that ARIA might not be anti-Aβ specific, and have seen evidence of ARIA in participants undergoing an anti-TREM2 antibody clinical trial, suggesting that the increased neuroinflammation, regardless of antibody target, may contribute to ARIA (Rogers, 2023).

### 2.3 APOE^ε4^, Aβ deposition, and CAA

In humans, post-mortem studies have shown that autopsied brains of APOE^ε4^ carriers contain more Aβ plaques. APOE^ε4^ is one of the only major genetic risk factors for cerebral amyloid angiopathy (CAA) (Rannikmae et al., 2014; Marini et al., 2019; Greenberg et al., 2020). CAA is primarily found in arteries and arterioles in the cortex and near the meninges (Vinters, 1987). In the absence of anti-Aβ immunotherapy, CAA can result in microbleeds, diminished cerebrovascular reactivity, and blood brain barrier communication breakdown (Greenberg et al., 2020). Across the Aβ fibril lifespan, the APOE^ε4^ allele influences Aβ seeding, as well as Aβ clearance from the brain, both contributing to overall increased pathology. Two critical studies identified APOE^ε4^ affects the early formation of Aβ fibril aggregation. One paper showed that ASO-mediated reduction of APOE prior to Aβ plaque deposition lessened Aβ plaque burden, however, this method was unable to mitigate existing Aβ plaque load (Huynh et al., 2017). Another paper utilized an inducible mouse model of astrocyte APOE^ε4^ or APOE^ε3^ expression and showed that APOE^ε4^ expression prior to Aβ aggregation caused worsened Aβ plaque deposition. Others that have tested the levels of APOE across alleles in association with Aβ plaque deposition have found that prior to Aβ plaque formation, APOE^ε4^ causes an increase in brain Aβ42 levels (Liu et al., 2017). Moreover, a subset of CAA falls under the domain of CAA-related inflammation, or CAA-ri, with rates that are highly elevated in APOE^ε4^ carriers (Kinnecom et al., 2007). These data suggest that APOE^ε4^ has a unique effect on Aβ protein levels as well as early and increased deposition in the brain compared to APOE^ε3^ controls. Further, mouse studies have also shown that the absence of APOE in a knock-out mouse model reduces CAA-related Aβ build up, substantiating the influence of APOE on vascular plaque load and CAA (Fryer et al., 2003; Miao et al., 2005).

CAA leads to reduced cerebrovascular integrity and function, leading to devastating consequences and ARIA, ultimately replacing much of the smooth muscle cell layer of the vasculature with amyloid. CAA can lead to spontaneous hemorrhagic events, however, the concept of anti-Aβ antibody induced CAA is somewhat novel and evidence for this is primarily from mouse model studies (Boche et al., 2008). CAA also results in reduced perivascular drainage, further increasing the buildup of Aβ in the brain (Hawkes et al., 2012). The underlying mechanisms by which anti-Aβ antibody drives CAA remain to be fully understood, however evidence does show that exacerbation of CAA was diminished when the anti-Aβ antibody was deglycosylated, diminishing its affinity for the Fcγ receptor.

Evidence suggest that CAA can occur due to impaired parenchymal Aβ plaque clearance, and some may postulate that this drastic increase in anti-Aβ antibody mediated clearance is triggering worsened CAA (Herzig et al., 2006). Studies on previous anti-Aβ antibody clinical trials showed that antibody-Aβ complexes are found essentially ‘stuck’ in the vasculature, similar to what is found during spontaneous CAA, suggesting that the immunotherapy is causing clearance of the complex which is impaired at the vessel level (Sakai et al., 2014). Further supporting this finding, there was worsened vessel ‘concentric splitting’, which is a splitting of the vessel into multiple layers, in the immunized participants, resulting damage may be caused by the mobility of APOE-Aβ complexes (Piazza et al., 2022). Interestingly, spontaneous CAA-related inflammation (CAA-ri), which is associated with apparent spontaneous ARIA, has been shown to cause increases in endogenous auto-anti-Aβ antibodies, suggesting a common mechanism between the immunotherapy and a dysregulated immune response that coalesce into ARIA (Piazza et al., 2013; Antolini et al., 2021; Zedde et al., 2023). Interestingly, in CAA-ri, one study showed that brains that had both AD and CAA pathologies, when compared to CAA alone, resulted in increased microglial response and more extreme ARIA. While similar in mechanism, the treatments for antibody-mediated ARIA is discontinuation of treatment or requires treatment with glucocorticosteroid, and such steroid therapy is used to treat CAA-ri (Cogswell et al., 2022). Prior to anti-Aβ antibody immunotherapy treatment, baseline evaluation via MRI for cortical microbleeds, a biomarker of CAA, is a red-flag for severely increased risk for ARIA (Cogswell et al., 2022).

## 3 Discussion: considerations moving forward

It is clear that there are consistent risk factors that contribute to increased ARIA risk. To mitigate the cerebrovascular damage while rolling out these drugs in clinical practice and in future clinical trials, there are several recommendations that are being utilized. The first being APOE genotyping. Each patient / participant should have their APOE status known prior to anti-Aβ antibody immunotherapy. While in clinical trial practice this may be confounding, in practice in the clinic for lecanemab, there should be an awareness on the administering clinician’s side of the risk that APOE^ε4^ carriage brings to the table for that patient. Second, there should be more stringent baseline MRI scans prior to treatment. Trained neuroradiologists should be reviewing and carefully scrutinizing baseline MRIs with an ARIA-specific protocol and template for reporting. Hopefully, in the near future, there will be automated AI generated programs that can detect ARIA-E and ARIA-H; however, careful examination of the patient’s scan could indicate their neutral or elevated risk for devastating ARIA during treatment. Our third suggestion is a clear plan of action to ensure that if ARIA symptomology does occur, therapy is suspended until ARIA resolves on imaging and symptoms are no longer apparent. Due to a small number of recent deaths reported in clinical trials, at least one of which was potentially caused by administration of tissue plasminogen activator (tPA, a common and often quick response to ischemic stroke), there should be an on-body indication of the ongoing treatment. A medical bracelet similar to ones worn by diabetics or epileptic persons would quickly indicate that no tPA should be administered without the neurologist’s input. As always, the administration of an anti-Aβ antibody is at the discretion of the physician, however, the appropriate use guidelines for each drug provide inclusion (must have Aβ positive tests) and exclusion (use of anti-coagulants) criteria that should be noted.

Anti-Aβ immunotherapies represent monumental progress in the treatment of Alzheimer’s disease, and the safety and efficacy has surpassed any previous attempts at a disease modifying therapy. While these treatments are highly effective in lowering brain Aβ burden, the disruptive effects on the cerebrovasculature in the form of ARIA cannot be ignored. Further mechanistic research into the direct mechanisms by which anti-Aβ antibodies caused cerebrovascular damage is necessary to facilitate equal treatment effects for all Alzheimer’s disease patients, including those carrying the high risk APOE^ε4^ gene.

## Author contributions

KF: Writing – original draft, Writing – review & editing. DW: Writing – original draft, Writing – review & editing.
